# Associations of fat and muscle mass with overall survival in men with prostate cancer: a systematic review with meta-analysis

**DOI:** 10.1038/s41391-021-00442-0

**Published:** 2021-08-21

**Authors:** Pedro Lopez, Robert U. Newton, Dennis R. Taaffe, Favil Singh, Laurien M. Buffart, Nigel Spry, Colin Tang, Fred Saad, Daniel A. Galvão

**Affiliations:** 1 Exercise Medicine Research Institute, Edith Cowan University, Joondalup, WA Australia; 2 School of Medical and Health Sciences, Edith Cowan University, Joondalup, WA Australia; 3 School of Human Movement and Nutrition Sciences, University of Queensland, St. Lucia, QLD Australia; 4 Department of Physiology, Radboud University Medical Center, Radboud Institute for Health Sciences, Nijmegen, The Netherlands; 5 Faculty of Medicine, University of Western Australia, Nedlands, WA Australia; 6 Department of Radiation Oncology, Sir Charles Gairdner Hospital, Perth, WA Australia; 7 Division of Urology and Urologic Oncology, Centre Hospitalier de l’Université de Montréal, Montreal, QC Canada

**Keywords:** Prostate cancer, Prognostic markers

## Abstract

**Background:**

To systematically review and analyse the associations between fat and muscle mass measures with overall survival in men with prostate cancer.

**Methods:**

A systematic search was conducted in CINAHL, Cochrane Library, EMBASE, PubMed, and Web of Science databases from inception to December 2020, while abstracts from the American Society of Clinical Oncology (ASCO), Clinical Oncology Society of Australia (COSA), and the American College of Sports Medicine (ACSM) conferences were searched from 2014 to 2020. Eligible articles examined the association of body composition measures, such as fat mass (e.g., fat mass, visceral adipose tissue (VAT), subcutaneous adipose tissue (SAT), and VAT/SAT) and muscle mass measures, with overall survival in prostate cancer patients at any treatment stage. The primary endpoint was overall survival. Random-effect meta-analysis was conducted for studies reporting multivariable or univariable analysis assessing the associations of fat mass measures (i.e., fat mass, VAT, SAT, VAT/SAT) and muscle mass measures with overall survival.

**Results:**

Sixteen cohort studies that comprised 4807 men with prostate cancer were included. Total adiposity (hazard ratio (HR) 0.98, 95% CI: 0.75–1.28, *p* = 0.888) and VAT (HR 1.03, 95% CI: 0.74–1.43, *p* = 0.873) were not significantly associated with overall survival, while higher subcutaneous adipose tissue levels were associated with higher survival (HR 0.68, 95% CI: 0.54–0.84, *p* = 0.001). Greater mortality risk was found in patients with localised (HR 1.91, 95% CI: 1.40–2.62, *p* < 0.001) and advanced disease (HR 1.43, 95% CI: 1.07–1.92, *p* = 0.020) presenting with low levels of muscle mass compared to those presenting with high levels.

**Discussion:**

These results indicate that although overall adiposity should be cautiously interpreted in regards to survival, high muscle mass and SAT, and low VAT/SAT ratio values are associated with overall survival in men with prostate cancer.

## Introduction

Prostate cancer is one of the most prevalent cancers worldwide, accounting for one in five new cancer cases in men [1]. Among the available treatments, androgen deprivation therapy (ADT) is commonly used alone or in combination with other forms of therapy to delay prostate cancer progression and improve survival in patients with advanced prostate cancer [2]. However, as a result of resistance to treatment [3, 4], altered metabolic profile and body composition impairments such as increased fat mass and reduced muscle mass [5, 6], patients are at an increased risk of both cancer and non-cancer related mortality with 5-year survival rates as low as 30% depending on health status and stage at the time of prostate cancer diagnosis [7].

Obesity is a potential predictor of mortality in men with prostate cancer [8, 9], affecting not only tumour biology [10] but also the outcomes of radical prostatectomy and radiation therapy [8, 11–13]. Significant associations between high body mass index (BMI; >30 kg m^−2^) and a 23% increased risk for all-cause mortality [9], or increases of 5 kg m^−2^ with a 20% increased risk of prostate cancer-specific mortality [8] were reported in previous investigations. However, the association of obesity with all-cause mortality is not consistent across all prostate cancer studies, with some studies challenging this relationship by presenting no significant association between higher BMI values and overall survival in this population [14, 15], or presenting an inverse relationship between obesity and survival [16]. This apparent obesity paradox may be related to the reliance on BMI since this measure does not differentiate lean from fat mass or visceral adipose tissue (VAT) and subcutaneous adipose tissue (SAT) [17, 18], masking the relationship of fat mass with overall survival in men with prostate cancer [19, 20]. Furthermore, sarcopenia or the loss of muscle mass has also been considered an important prognostic factor [6, 21–23], although its association with overall survival in men with prostate cancer is largely controversial depending on the cancer stage or phase of treatment [16, 24, 25]. Therefore, it remains to be determined if excess fat mass, reduced levels of muscle mass, or both treatment-related changes in body composition have an impact on overall survival in men with prostate cancer [20]. Determining these associations may potentially inform specific and tailored strategies to improve overall survival in this group of patients.

As a result, we investigated in this systematic review the role of body composition on overall survival in men with prostate cancer, analysing the associations of low muscle mass and high fat mass as prognostic factors. In addition, a range of possible clinical (i.e., localised vs. advanced disease) and methodological (i.e., definition of cut-off values for muscle mass, depots of fat mass and controlling for BMI in multivariable analysis) variables that may affect the associations of body composition with overall survival were examined by subgroup analyses.

## Methods

### Study selection procedure

A systematic search was conducted in the following electronic databases: CINAHL, Cochrane Library, EMBASE, PubMed and Web of Science from inception to December 2020. The search strategy is presented in the Supplementary eAppendix 1. In addition, we also performed a manual search of the reference lists provided in the selected papers as well as in abstracts from the American Society of Clinical Oncology, Clinical Oncology Society of Australia and the American College of Sports Medicine conferences from 2014 to 2020. All procedures were undertaken in accordance with the Preferred Reporting Items for Systematic Reviews and Meta-Analyses statement [26, 27] and based on the minimum criteria established by the Cochrane Back Review Group [28], with registration at the international prospective register of systematic reviews (PROSPERO identifier: CRD42020218736).

This review included published articles and conference abstracts [29] of studies evaluating the association of body composition measures, such as fat mass (e.g., fat mass, VAT, SAT and VAT/SAT) and muscle mass measures, with overall survival in prostate cancer patients at any treatment stage. The primary and only outcome for this review was overall survival, defined as the time in months of death by any cause. The exclusion criteria were: (1) studies involving mixed cancer patients without specific information on the results for prostate cancer patients; (2) studies not including or reporting on the specific outcomes for this review, or did not include sufficient information such as hazard ratios (HR) and 95% confidence intervals (CI) for overall survival analysis; (3) studies evaluating specific interventions for body composition such as nutrition or exercise; and (4) written in a language other than English. In the search strategy, titles and abstracts were first independently evaluated following the eligibility criteria. When abstracts did not provide sufficient information, they were selected for full-text evaluation. In addition, authors were contacted for further information when necessary. Eligibility was assessed independently in duplicate (PL and FS), with differences resolved by consensus.

### Data extraction

Data extraction was performed via a standardised form. Clinical and methodological information were extracted from the included studies such as cancer stage and treatment, number of participants at baseline, geographical region, age and BMI at baseline, fat and muscle mass assessments (i.e., method of assessment, location and cut-off values), follow-up period, HR for overall survival with their associated dispersion values such as 95% CI or standard errors (SE) from univariable and multivariable analyses, when available, and the number of covariates included in the multivariable models.

### Study quality assessment

The study quality assessment was evaluated according to the Newcastle-Ottawa Quality Assessment Scale (NOS) for cohort studies [30]. The NOS consists of eight items related to representativeness of the exposed cohort, comparability based on the study design or analysis and assessment of outcome and adequacy of follow-up with a total maximum score of 9 [30]. Studies were assessed by the following items: (1) Representativeness of the exposed cohort; (2) Selection of the non-exposed cohort; (3) Ascertainment of exposure; (4) Demonstration that outcome of interest was not present at start of study; (5) Comparability of cohorts on the basis of the design or analysis; (6) Assessment of outcome; (7) Was follow-up long enough for outcomes to occur; (8) Adequacy of follow up of cohorts. The study quality assessment for all included studies were performed independently by two reviewers (PL and FS) with disagreements resolved by consensus.

### Data analysis

Data from the associations of body composition with overall survival such as HR and their associated dispersion values were pooled using inverse variance random-effects models. These values were extracted from univariable and multivariable models and log-transformed to be included in further analyses. Analyses were conducted for studies reporting multivariable or univariable analysis and subgroup analyses were provided for the following: (1) removing outliers; (2) for prostate cancer subgroups (stage or phase of treatment); (3) previously defined or median cut-off values for muscle mass outcomes; (4) specific depots of fat and (5) studies using BMI as a covariate or not in the multivariable models. A *p* value of ≤0.05 was considered statistically significant. Forest plots were generated to present the results for multivariable and univariable analysis of fat and muscle mass. Heterogeneity between studies was assessed by using the *I*^2^ statistic and the *p* value from *χ*^2^-based Cochran’s *Q* test with a high heterogeneity defined by a threshold *p* value of 0.1 or *I*^2^ value greater than 50% [31]. We examined outliers using sensitivity analysis by omitting one study at a time. To check for publication bias, contour-enhanced funnel plots of log HR against its SE were generated and explored using Egger’s regression asymmetry test when more than ten studies were available [32]. Analyses were conducted using the Review Manager (RevMan) software from the Cochrane Collaboration (version 5.4, Copenhagen: The Nordic Cochrane Centre) and the package ‘meta’ from R (R Core Team, 2020).

## Results

### Studies included and characteristics

**Fig. 1:**
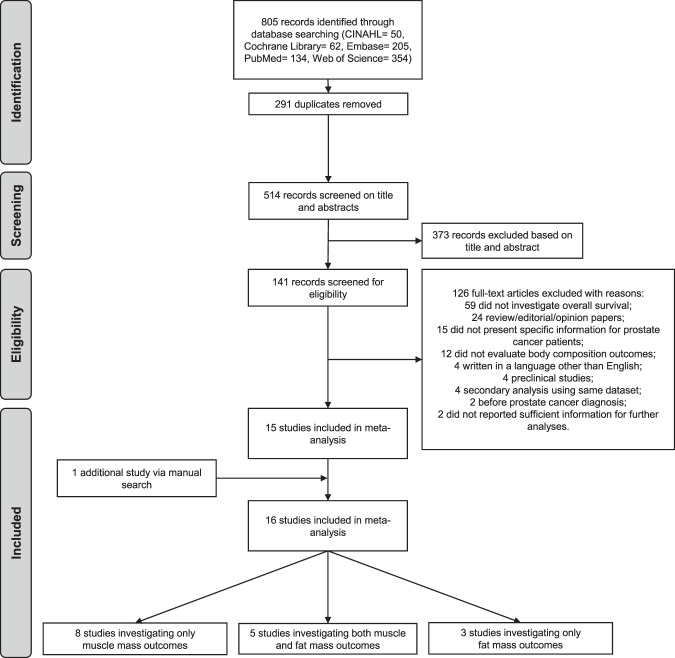
Flow chart. Flow chart of study selection process.

Of the 805 retrieved studies, 514 potential records were retained for screening after duplicate removals. Of these, 373 were excluded due to their irrelevance to the research question and 141 articles were deemed eligible and undertaken for review (Fig. 1 and Supplementary eAppendix 2). A total of 16 cohort studies undertaking retrospective analyses [16, 24, 25, 33–45] were included in the primary analysis. During the eligibility assessment, six additional studies [46–51] were initially selected and authors contacted given the lack of specific information on the results for prostate cancer patients. Responses were not obtained and, as a result, these studies were not included in our review.

The characteristics of the individual studies are presented in Tables 1 and 2. In summary, a total of 4807 prostate cancer patients with a median age of 69.0 years (interquartile range (IQR): 67.2–71.3) and BMI of 26.6 kg m^−2^ (IQR: 24.3–28.7) participated in the included studies. All studies except one derived fat and muscle mass measures from CT scans [16, 24, 25, 34–45]. Most studies (*n* = 11) included advanced prostate cancer patients (e.g., metastatic, castration-resistant or metastatic castration-resistant patients) [16, 24, 34, 35, 38, 39, 41–45], and the majority of patients received treatments such as radiotherapy [33, 36–43] and ADT [33, 35–37, 40–44] (*n* = 9 for both), followed by surgery [25, 33, 36, 37, 39, 42, 43] and chemotherapy [34, 35, 39, 41–43, 45] (*n* = 7 for both), and novel hormonal agents such as abiraterone and enzalutamide (*n* = 1) [24]. Regarding the quality assessment, the median overall score was seven out of nine ranging from 4 to 9 pts. The quality assessment of individual studies is presented in eTable 1 (Supplementary material).

**Table 1:** Study characteristics: country, patient characteristics, sample size, cancer treatment and study follow-up.

Author (ref.)	Country	Patient characteristics	Sample size	Treatment	Follow-up
Antoun et al. [24]	France	Patient subgroup: mCRPCa patients Age: mean of 69 ± 8 years BMI: 26.1 ± 4.0 kg m^−2^	*n* = 127	Enzalutamide, abiraterone acetate and prednisone or placebo	Median of 45 months (95% CI: 31–47 months)
Buttigliero et al. [33]	Italy	Patient subgroup: PCa patients treated with androgen deprivation therapy and without bone metastasis Age: mean of 73 years (ranging from 44 to 83) BMI: median of 25 kg m^−2^ (ranging from 19 to 39)	*n* = 53	Radical prostatectomy and/or radiation therapy and androgen deprivation therapy	Median of 76 months
Wu et al. [34]	USA	Patient subgroup: Metastatic PCa patients treated with docetaxel Age: NR BMI: NR	*n* = 333	Chemotherapy	NR
Cushen et al. [35]	Ireland	Patient subgroup: mCRPCa patients Age: mean of 69 ± 8.4 years BMI: 27.8 ± 4.3 kg m^−2^	*n* = 63	Androgen deprivation therapy, and chemotherapy	72 months
McDonald et al. [36]^a^^,b^	USA	Patient subgroup: PCa patients undergoing definitive external beam radiotherapy and/or brachytherapy Age: mean of 65.1 ± 7.9 years BMI: 29.0 ± 5.52	*n* = 652	Prostatectomy, radiation therapy, and androgen deprivation therapy	Median of 6.6 years
Mason et al. [37]	USA	Patient subgroup: PCa patients undergoing radical prostatectomy (open or robot assisted) Age: mean of 61.8 ± 7.1 years BMI: 28.6 ± 4.1 kg m^−2^	*n* = 698	Prostatectomy, radiation therapy and androgen deprivation therapy	Median of 6.0 years
Zakaria et al. [38]	USA	Patient subgroup: PCa patients with spinal column metastasis Age: mean of 72.8 ± 8.5 years BMI: NR	*n* = 92	Bisphosphates, antiangiogenic drugs, radiation therapy	NR
Ohtaka et al. [39]	Japan	Patient subgroup: CRPCa patients Age: median of 70 years (ranging from 65 to 76) BMI: median of 24.0 kg m^−2^ (ranging from 21.3 to 25.9)	*n* = 77	Prostatectomy, radiation therapy and chemotherapy	Median of 499 days (IQR: 333–790 days)
Pak et al. [25]	South Korea	Patient subgroup: PCa patients undergoing radical prostatectomy Age: mean of 66.1 years BMI: 24.7 kg m^−2^	*n* = 1020^c^	Prostatectomy	Median of 94.3 months
Di Bella et al. [40]	USA	Patient subgroup: PCa patients treated with primary external beam radiotherapy or brachytherapy. Age: mean of 63.9 ± 6.7 years BMI: median of 29.0 kg m^−2^ (IQR: 25.7–33.4)	*n* = 401	Androgen deprivation therapy, and radiation therapy	Median of 9.3 years (IQR: 7.3–10.6 years)
Ikeda et al. [41]	Japan	Patient subgroup: mHSPCa Age: median of 73 years (IQR: 66–78) BMI: median of 22.2 kg m^−2^ (IQR: 20.0–23.9)	*n* = 197	Androgen deprivation therapy, chemotherapy and radiation therapy	Median of 39 months (IQR: 25–61 months)
Lee et al. [42]	South Korea	Patient subgroup: mCRPCa patients Age: median of 70 years (IQR: 65–76) BMI: 24.4 kg m^−2^ (ranging from 22.5 to 26.3)	*n* = 411	Prostatectomy, radiotherapy, androgen deprivation therapy and chemotherapy	NR
Pak et al. [43]	South Korea	Patient subgroup: CRPCa patients Age: 68.3 years BMI: 23.6 kg m^−2^	*n* = 230	Prostatectomy, radiotherapy, androgen deprivation therapy and chemotherapy	Median of 21.3 months
Sasaki et al. [44]	Japan	Patient subgroup: Hormone-naïve men with advanced PCa Age: median of 71 years (ranging from 49 to 93) BMI: median of 22.7 kg m^−2^ (ranging from 15.9 to 31.7)	*n* = 85	Prostatectomy, androgen deprivation therapy	Median of 50.6 months (ranging from 8 to 151 months)
Stangl-Kremser et al. [45]	Austria	Patient subgroup: CRPCa patients Age: median of 68.8 years (IQR: 64.6–75.0) BMI: median of 27.0 kg m^−2^ (IQR: 25.2–29.8)	*n* = 186	Chemotherapy	Median of 24.1 months (IQR: 12.8–40.8)
Xu et al. [16]	USA	Patient subgroup: CRPCa patients Age: median of 71.5 years (IQR: 64.9–76.1) BMI: median of 28.8 kg m^−2^ (ranging from 17.8 to 54.7)	*n* = 182	NR	Median of 33.9 months (IQR: 20.4–55.2 months)

*BMI* body mass index, *CRPCa* castration-resistance prostate cancer, *IQR* interquartile range, *NR* not reported, *mCRPCa* metastatic castration-resistance prostate cancer, *mHSPCa* metastatic hormone-sensitive prostate cancer, *PCa* prostate cancer.

^a^Data derived from conference abstract.

^b^Data checked by accompanied paper.

^c^Sample size derived from highest and lowest quartile stratification level.

**Table 2:** Study characteristics: body composition assessment, location, outcomes and cut-off values, multivariate model covariates, total of deaths and median overall survival.

Author (ref.)	Body composition assessment, timepoint and location	Body composition outcomes and cut-off values	Multivariate model	Overall survival
Antoun et al. [24]	CT scans of L3 Timepoint: NR	Sarcopenia BMI < 25 kg m^−2^ and SMM index <43 cm^2^ m^−2^ OR BMI ≥ 25 kg m^−2^ and SMM index <53 cm^2^ m^−2^ OR BMI ≥ 30 kg m^−2^ and SMM index ≥53 cm^2^ m^−2^ SMM index^a^ SMM index <45 cm^2^ m^−2^ VAT index^a^ VAT index ≥52.2 cm^2^ m^−2^ SAT index^a^ SAT index ≥51.7 cm^2^ m^−2^	Age, BMI, NRS pain, ECOG performance status, LDH, presence of visceral metastases, PSA, haemoglobin, albumin, alkaline phosphatase, sarcopenia, VAT, SAT	Total deaths: 101 (80%); Median 3-year OS: 16 months (95% CI: 12–19 months)
Buttigliero et al. [33]	DXA whole-body measurements Timepoint: Before starting AST	LBM^a^ Below the median value at baseline FBM^a^ Above the median value at baseline	-	Total deaths: 22 (44%); Median OS: NR
Wu et al. [34]	CT scans of L3-4 Timepoint: Within 1 month from the initiation of docetaxel	VAT/SAT ratio^a^ Above the median value at baseline	Time after diagnosis, age, race, Gleason score, alkaline phosphatase, visceral fat-to-muscle area ratio, BMI, weekly regimen, chemotherapy dosage	Total deaths: 240 (72.1%) Median OS: 21.1 months (95% CI: 17.8–24.4)
Cushen et al. [35]	CT scans of L3 Timepoint: NR	SMM index BMI ≥ 25 kg m^−2^ and SMM index <53 cm^2^ m^−2^ OR BMI < 25 kg m^−2^ and SMM index ≤43 cm^2^ m^−2^ VAT index VAT index ≥58.7 cm^2^ m^−2^ SAT index SAT index ≥55.3 cm^2^ m^−2^	Anaemia, BMI, VAT	Total deaths: 37 (58.7%); Median OS: 17.3 months (ranging from 14.3 to 20.4 months)
McDonald et al. [36]^b^	CT scans of L4-5 Timepoint: Within 3 months of radiotherapy	Psoas muscle index Psoas muscle index <7.5 cm^2^ m^−2^	Age, comorbidity, Prostate cancer risk grouping, race, AST	Total deaths: NR; Median OS: NR
Mason et al. [37]	CT scans of L3 Timepoint: Within 6 months before prostatectomy	SMM index SMM index <55 cm^2^ m^−2^	Age, Gleason score, tumour stage, lymph node, PSA, positive margins, ADT	Total deaths: 50 (7.1%); Median OS: NR
Zakaria et al. [38]	CT scans of L4 Timepoint: NR	Average psoas muscle size^a^	Age, number of levels (single vs. multiple), bisphosphonates, antiangiogenic drugs	Total deaths: 77 (84%) Median OS: 124 days (95% CI: 98–197)
Ohtaka et al. [39]	CT scans of L3 Timepoint: NR	Psoas muscle index Psoas muscle index <5.7 cm^2^ m^−2^	Albumin, neutrophil-lymphocyte ratio, LDH, haemoglobin, alkaline phosphatase	Total deaths: 35 (45%); Median OS: 19.6 months in patients treated with docetaxel and 16.7 months in patients treated with mitoxantrone.
Pak et al. [25]	CT scans of L3 Timepoint: Prior prostatectomy	Psoas muscle index^a^ Psoas muscle index = 4.74 cm^2^ m^−2^ (IQR: 4.28–5.06)	BMI	Total deaths: NR; Median OS: NR
Di Bella et al. [40]	CT scans of L4-5 Timepoint: At the time of radiation therapy	Visceral fat area^a^ Visceral fat area ≥287.32 cm^2^ Subcutaneous fat area^a^ Subcutaneous fat area ≥36.44 cm^2^	Age, race, year, biopsy grade group, PSA, clinical stage, ADT	Total deaths: 138 Median OS: NR
Ikeda et al. [41]	CT scans of L3 Timepoint: Within 2 months before starting AST	SMM index BMI ≥ 25 kg m^−2^ and SMM index <53 cm^2^ m^−2^ OR BMI < 25 kg m^−2^ and SMM index ≤43 cm^2^ m^−2^	BMI, LDH, Gleason score, Latitude risk classification	Total deaths: 80 (40.6%); Median OS: NR
Lee et al. [42]	CT scans of L3 Timepoint: At the time of castration-resistance diagnosis	SMM index^a^ SMM index <45.2 cm^2^ m^−2^	Age, BMI	Total deaths: NR; Median OS: 19 months for low SMM index and 24 for high SMM index
Pak et al. [43]	CT scans of L3 Timepoint: Before starting first-line treatments for castration-resistance	SMM index^a^ SMM index <49.9 cm^2^ m^−2^ VAT index^a^ VAT index ≥59.4 cm^2^ m^−2^ SAT index^a^ SAT index ≥48.2 cm^2^ m^−2^	Age, BMI, PSA, ECOG performance status, SMM index, bone metastasis, solid organ metastasis	Total deaths: NR Median OS: 16.9 months for low SMM index and 24.1 months for high SMM index
Sasaki et al. [44]	CT scans at the level of the umbilical position Timepoint: NR	VAT area VAT area ≥100 VAT/SAT area ratio VAT/SAT area ratio ≥1	-	Total deaths: 36 (42.3%); Median OS: NR
Stangl-Kremser et al. [45]	CT scans of L3 Timepoint: Before initiation of chemotherapy	SMM index BMI ≥ 25 kg m^−2^ and SMM index <53 cm^2^ m^−2^ OR BMI < 25 kg m^−2^ and SMM index ≤43 cm^2^ m^−2^ Skeletal muscle volume index^a^ Skeletal muscle volume index <28.7 kg VAT index^a^ VAT index ≥68.0 cm^2^ m^−2^ SAT index^a^ SAT index ≥64.1 cm^2^ m^−2^ VAT/SAT ratio^a^ NR	Liver metastasis, BMI, LDH, VAT/SAT ratio	Total deaths: 93 (50%) Median OS: 26.2 months (IQR: 13.7–42.4)
Xu et al. [16]	CT scans of L3 Timepoint: NR	SMM index BMI ≥ 25 kg m^−2^ and SMM index <53 cm^2^ m^−2^ OR BMI < 25 kg m^−2^ and SMM index ≤43 cm^2^ m^−2^	Age, BMI, Charlson Comorbidity Index, race, metastasis, hormone-sensitive	Total deaths: NR Median OS: 50.6 ± 6.1 months for sarcopenia and 55.5 ± 5.8 months for patients without sarcopenia

*ADT* androgen deprivation therapy, *BMI* body mass index, *CT* computerised tomography, *ECOG* Eastern Cooperative Oncology Group, *FBM* whole-body fat mass, *LDH* lactate dehydrogenase, *NR* not reported, *NRS* numerical rating scale for pain assessment, *PSA* prostate-specific antigen, *SAT index* subcutaneous adipose tissue index, defined as SAT normalised to height squared, *SMM index* skeletal muscle mass index, defined as SMM normalised to height squared, *VAT index* visceral adipose tissue index, defined as VAT normalised to height squared.

^a^Based on the median values of the sample.

^b^Data checked by accompanied paper.

### Fat mass and overall survival

**Fig. 2:**
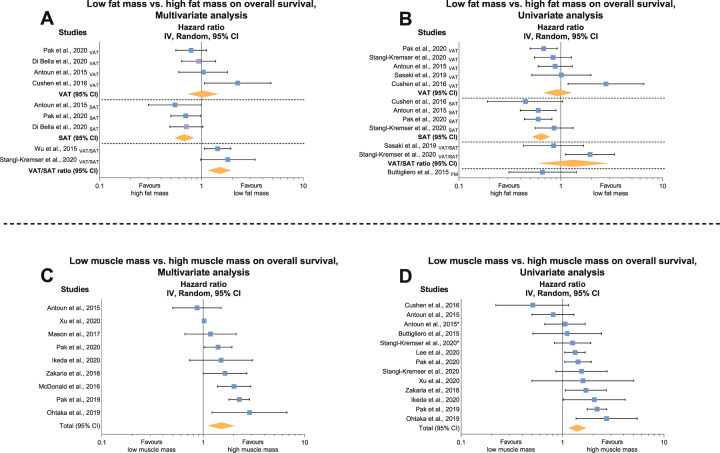
Random-effects meta-analysis. Association of low and high levels of VAT, SAT and VAT/SAT ratio (**A**, **B**) and muscle mass (**C**, **D**) with overall survival in men with prostate cancer. Analyses derived from multivariable and univariate models were presented in **A**, **C** and **B**, **D**, respectively. Higher VAT/SAT ratio indicates poorer overall survival. Overall effects analyses conducted with inverse variance random-effects meta-analysis. Squares represent study-specific estimates; diamonds represent pooled hazard ratios estimates of random-effects meta-analysis. *Study-specific estimate based on median values derived from skeletal muscle mass index and skeletal muscle volume indexes; FM fat mass, SAT subcutaneous adipose tissue, VAT visceral adipose tissue.

Eight studies [24, 33–35, 40, 43–45] comparing high vs. low levels of fat mass on overall survival were included in the analysis, with six studies examining VAT (cut-off values reported: 52.2 cm^2^ m^−2^ [23], 58.7 cm^2^ m^−2^ [35], 59.4 cm^2^ m^−2^ [43], 68.0 cm^2^ m^−2^ [45], 100.0 cm^2^ [44] and 287.3 cm^2^ [40]) [24, 35, 40, 43–45], five studies examining SAT (cut-off values reported: 48.2 cm^2^ m^−2^ [43], 51.7 cm^2^ m^−2^ [24], 55.3 cm^2^ m^−2^ [35], 64.1 cm^2^ m^−2^ [45] and 36.4 cm^2^ [40]) [24, 35, 40, 43, 45], three studies examining VAT/SAT ratio (with one study reporting a cut-off of 1.0 [44]) [34, 44, 45] and one study examining whole-body fat mass [33]. Given that six studies [24, 34, 35, 40, 43, 45] undertook multivariable models controlling for BMI (median number of covariates of 7.0, ranging from 2 to 12; with two studies also controlling for muscle mass measures [24, 43]), the results from the meta-analysis provided no differences in overall survival (HR 0.98, *p* = 0.888; Table 3) in a sample of 1697 prostate cancer patients. The heterogeneity was *I*^2^ = 70%. Patients presenting with high levels of SAT are at an advantage for overall survival compared to those presenting with low SAT levels (HR 0.68, 95% CI: 0.54–0.84; Fig. 2A), while analysis for VAT/SAT ratio provided a 50% greater mortality risk (HR 1.50, 95% CI: 1.15–1.97; Fig. 2A) to patients presenting high levels compared to those presenting with low levels. No difference was observed regarding VAT (*p* = 0.873; Fig. 2A), between results derived VAT and VAT/SAT (*χ*^2^ = 3.1; *p* = 0.080), while VAT and SAT (*χ*^2^ = 4.0; *p* = 0.045) and SAT and VAT/SAT (*χ*^2^ = 19.5; *p* < 0.001) were significantly different (Table 3). Differences were also not observed between patients with localised and advanced disease (*χ*^2^ = 1.2; *p* = 0.275) or for studies controlling for BMI (*χ*^2^ = 1.2; *p* = 0.273). In the univariable analysis, a 23% survival advantage was found after removing the study of Stangl-Kremser et al. [45] considered an outlier for the overall effect (HR 0.77, 95% CI: 0.64–0.92; Table 3), while the direction of the results was maintained for all subgroup analyses (*p* = 0.061–0.438; Table 2 and Fig. 2B) without differences between covariates. No publication bias was found (*p* = 0.146; Supplementary eFig. 1A).

**Table 3:** Overall and subgroup analyses of high fat mass vs. low fat mass on overall survival in prostate cancer patients.

Outcomes	No. of comparisons	Sample size	Main effect			Subgroup differences	
			HR (95% CI)	*I*^2^	*p* value	*χ*^2^	*p* value
*Multivariable analysis*							
Overall effect	8	1,697	0.98 (0.75–1.28)	70%	0.888	–	–
Overall effect without outlier	–	–	–	–	–	–	–
Population subgroups							
Advanced disease	7	1,296	1.05 (0.75–1.48)	75%	0.769	1.2	0.273
Localised disease	2	802	0.82 (0.63–1.08)	15%	0.166		
Outcome subgroups							
VAT	4	821	1.03 (0.74–1.43)	52%	0.873	4.0^a^	0.045
SAT	3	758	0.68 (0.54–0.84)	0%	0.001	3.1^b^	0.080
VAT/SAT ratio	2	519	1.50 (1.15–1.97)	0%	0.003	19.5^c^	<0.001
Multivariate models controlling for BMI							
Yes	7	1,296	1.05 (0.75–1.48)	75%	0.769	1.2	0.273
No	2	802	0.82 (0.63–1.08)	15%	0.166		
*Univariable analysis*							
Overall effect	12	744	0.84 (0.67–1.05)	60%	0.126	–	–
Overall effect without outlier	11	744	0.77 (0.64–0.92)	39%	0.005	–	–
Population subgroups							
Advanced disease	11	691	0.85 (0.67–1.08)	63%	0.189	–	–
Localised disease	1	53	0.66 (0.31–1.43)	–	–		
Outcome subgroups							
VAT	5	691	0.93 (0.67–1.30)	59%	0.678	3.5^a^	0.061
SAT	4	606	0.64 (0.52–0.79)	0%	<0.001	0.6^b^	0.438
VAT/SAT ratio	2	271	1.32 (0.59–2.96)	42%	0.503	2.8^c^	0.092
FM	1	53	0.66 (0.31–1.43)	–	–	–	–

*BMI* body mass index, *FM* fat mass, *HR* hazard ratio, *I*^*2*^ indicator of heterogeneity (%), *SAT* subcutaneous adipose tissue, *VAT* visceral adipose tissue.

^a^VAT vs. SAT.

^b^VAT vs. VAT/SAT.

^c^SAT vs. VAT/SAT.

### Muscle mass and overall survival

Thirteen studies [16, 24, 25, 33, 35–39, 41–43, 45] comparing low vs. high levels of muscle mass on overall survival were included in the analysis, with eight studies examining skeletal muscle mass index (cut-off values reported: 43.0 or 53.0 cm^2^ m^−2^ [16, 35, 41, 45], 45.0 cm^2^ m^−2^ [24], 45.2 cm^2^ m^−2^ [22], 49.9 cm^2^ m^−2^ [43] and 55.0 cm^2^ m^−2^ [37]) [16, 24, 35, 37, 41–43, 45], three studies examining psoas muscle index (cut-off values reported: 4.7 cm^2^ m^−2^ [25], 5.7 cm^2^ m^−2^ [39] and 7.5 cm^2^ m^−2^ [36]) [25, 36, 39], one study examining average psoas muscle size [38] or skeletal muscle volume index (cut-off value reported: 28.7 kg [45]). Meta-analysis involving data derived from multivariable models (median number of covariates of 5, ranging from 1 to 12; with two studies also controlling for fat mass measures [24, 43]) resulted in 50% greater mortality risk (HR 1.50, 95% CI: 1.11–2.05; Table 4 and Fig. 2C) for patients presenting with low levels of muscle mass compared to those presenting with high levels in a sample of 3275 men with prostate cancer. The study of Xu et al. [16] was considered an outlier in the analysis. After adjustment, the meta-analysis resulted in a HR of 1.63 (95% CI: 1.27–2.08; Table 3) with a heterogeneity *I*^2^ = 58%. The results were maintained in the subgroup analyses (HR 1.43–1.91, *p* = <0.001–0.036) except for studies controlling for BMI, which approached statistical significance (HR 1.48, 95% CI: 0.98–2.26, *p* = 0.060). Similarly, results were similar in univariable model analyses (HR 1.31–1.40; *p* = 0.002–0.004; Table 4 and Fig. 2D) except for those using previously defined cut-off values (*p* = 0.271; Table 3). No differences were observed between covariates in either multivariable or univariable models (*p* = 0.184–0.974). No publication bias was found (*p* = 0.301; Supplementary eFig. 1B).

**Table 4:** Overall and subgroup effects of low muscle mass vs. high muscle mass on overall survival in prostate cancer patients.

Analysis	No. of comparisons	Sample size	Main effect			Subgroup differences	
			HR (95% CI)	*I*^2^	*p* value	*χ*^2^	*p* value
*Multivariate analysis*							
Overall effect	9	3275	1.50 (1.10–2.05)	88%	0.009	–	–
Overall effect without outlier	8	3093	1.63 (1.27–2.08)	58%	<0.001	–	–
Population subgroups							
Advanced disease^a^	5	723	1.43 (1.07–1.92)	34%	0.020	1.8	0.184
Localised disease	3	2370	1.91 (1.40–2.62)	52%	<0.001		
Previously defined cut-off values							
Yes^a^	5	1751	1.50 (1.01–2.22)	56%	0.036	0.4	0.532
No	3	1342	1.77 (1.27–2.48)	67%	<0.001		
Multivariate models controlling for BMI							
Yes^a^	4	1574	1.48 (0.98–2.26)	77%	0.060	0.5	0.502
No	4	1519	1.77 (1.32–2.36)	17%	<0.001		
*Univariate analysis*							
Overall effect	13	2638	1.40 (1.13–1.72)	64%	0.002	–	–
Overall effect without outlier	12	1618	1.31 (1.09–1.58)	39%	0.004	–	–
Population subgroups							
Advanced disease	11	1565	1.32 (1.09–1.61)	44%	0.005	0.7	0.413
Localised disease	2	1073	1.74 (0.93–3.25)	62%	0.083		
Previously defined cut-off values							
Yes	6	832	1.32 (0.80–2.17)	67%	0.271	0.0	0.974
No^a^	6	1099	1.33 (1.15–1.54)	0%	<0.001		

*BMI* body mass index, *HR* hazard ratio, *I*^*2*^ indicator of heterogeneity (%).

^a^Adjustment after sensitivity analysis omitting one study at a time.

## Discussion

In this review we examined the role of fat and muscle mass on survival in men with prostate cancer. The main findings of our study were: (1) although overall fat mass was not a prognostic factor in men with prostate cancer, high levels of subcutaneous fat and low levels of VAT/SAT were associated with a 32% and 50% survival advantage, respectively, in patients at advanced stages of the disease; and (2) patients presenting with low muscle mass levels are at ~50% increased risk of mortality compared to those presenting with high levels regardless of the cancer stage or methodological characteristics. These results are clinically relevant and indicate the importance of muscle mass in particular during the course of therapy given the substantial impact on overall survival of patients with prostate cancer.

Although obesity and the resulting metabolic environment are deemed important factors for biochemical recurrence, metastatic disease and mortality in men with prostate cancer [8, 9], our finding is that total adiposity is not associated with overall survival in prostate cancer patients. Interestingly, the reasons for this particular outcome may be related to the metabolic differences between SAT and VAT [52], with subcutaneous and visceral depots of fat exerting conflicting effects on overall survival in prostate cancer patients. For example, researchers have suggested that VAT is closely associated with inflammatory cytokines (e.g., interleukin-6 and tumour necrosis factor-alpha) which may potentially affect the tumour microenvironment [10, 52], while subcutaneous tissue-derived factors such as leptin may act in contrast by increasing insulin sensitivity and lipid metabolism, thereby, effectively improving survival [52–55]. Another potential explanation for the different findings reported previously [8, 9] and this study may be related to cancer cachexia [19]. This phenomenon may mislead the association of obesity with cancer progression or mortality given the unintentional weight loss that can occur during cancer treatment or even before the cancer detection (i.e., reverse causation) in obese cancer patients [19]. Thus, the assessment of BMI alone at the time of cancer may not inform whether prostate cancer patients have been obese before diagnostic, precluding us to specifically observe the influence of obesity on cancer survival in prostate cancer patients. Finally, our data on fat mass and overall survival were derived from studies mostly with advanced prostate cancer patients (i.e., metastatic and castration-resistant patients) and this may explain the difference between our findings and a previous study indicating significant associations between BMI and weight gain with prostate cancer outcomes in nonmetastatic patients [9]. Our results are in line with previous studies concerning the prognostic value of different depots of fat mass in cancer patients [24, 52, 56] and may indicate the necessity to cautiously interpret total adiposity in this group of patients, as different levels of obesity and depots of fat are influencing overall survival in opposite ways [57]. Therefore, the utilisation of the VAT/SAT ratio may be a good strategy to avoid such conflicting effects derived from different depots of fat. For example, in a previous study [34] high levels of VAT/SAT ratio were significantly associated with shorter survival in normal weight prostate cancer patients, although this relationship was not observed in overweight or obese patients. Consequently, more research is required to elucidate the physiological value of VAT/SAT ratio on overall survival. Moreover, although high levels of VAT did not significantly increase the risk of mortality in our analysis, previous studies have indicated the association with radical prostatectomy and radiation therapy outcomes [40, 58] increasing surgical and recurrence risks, respectively, as well as increased risk of cardiovascular and metabolic disease [59]. Thus, more studies are necessary to elucidate the indirect or direct role of VAT on overall survival in men with prostate cancer.

Contrary to the results regarding total adiposity, a high level of muscle mass was associated with improved overall survival in prostate cancer patients regardless of treatment stage or methodological characteristics. This may be related to the numerous benefits of muscle mass on metabolic health such as regulating and mobilising natural killer cells into the tumour, or even altering other biomarkers associated with the tumour biology [60, 61]. In addition, crosstalk between muscle and other organs has also emerged as a potential mechanism by which the musculoskeletal system supresses cancer growth and therefore increases overall survival in men with prostate cancer [60, 61]. Moreover, the present findings on muscle mass are in accordance with several other studies indicating a relationship between sarcopenia and survival in cancer patients [21–23] suggesting the importance of improving or maintaining muscle mass in this population before and during treatment [62]. For example, several trials have demonstrated the benefits of exercise, specifically resistance-based exercise programmes (i.e., anabolic exercise) increasing muscle mass during and following ADT [63–66], as well as preserving muscle mass in high-grade patients [67]. Therefore, the findings from our review are clinically meaningful indicating that muscle mass is an important prognostic factor for men with prostate cancer regardless of cancer stage. Also, accrual or maintenance of muscle mass through prescribed and tailored exercise, specifically resistance-based exercise, undertaken before, during and following cancer treatment would be beneficial in this population to effectively increase the chances of overall survival.

The strengths of the present study are: (1) a relatively large number of studies (*n* = 16) with up to 4807 prostate cancer patients included; (2) the assessment of both univariable and multivariable models; and (3) subgroup analyses based on different clinical and methodological characteristics. However, there are also some limitations which are worthy of comment. First, only cohort studies undertaking retrospective analyses were included in our review and this precludes determining causality of body composition changes such as increase in muscle mass and reduction in fat mass on overall survival. Future prospective analyses are necessary to improve current knowledge by indicating if interventions targeting fat and muscle mass are able to improve overall and disease-specific survival as well as recurrence in men with prostate cancer. In addition, researchers used different definitions for sarcopenia such as the skeletal muscle index, psoas muscle or median values, while no definition for adiposity measures was used, leading to high heterogeneity within the models. Sarcopenia has become a topic of great interest in oncology [68]; however, poorly understood given the lack of reporting on assessment characteristics and definitions. For example, although studies had reported the time of body composition assessment [25, 33, 34, 36, 37, 40–43, 45], the high heterogeneity and lack of standardisation could misclassify prostate cancer patients with obesity or sarcopenia and, therefore, mask the impact of these outcomes on overall survival. Future research should better inform definitions (i.e., cut-off values and rationale) and time of assessments, and information about clinical factors associated with muscle mass accrual or maintenance in this group of patients. This will improve the assessment of sarcopenia as well as provide information about its interaction with obesity on overall survival in this group of patients and assist future systematic reviews and meta-analyses. Nonetheless, subgroup analysis was undertaken based on previously defined methods to identify sarcopenia vs. median values to minimise such bias, with the results largely maintained, although the same was not conducted for fat mass given the lack of previous definitions. Finally, data concerning the association of fat mass and muscle mass measures with prostate cancer-specific and cardiovascular mortality as well as specific information about deaths are limited. Therefore, it is not possible to account for deaths directly or indirectly related to prostate cancer treatment comorbidities (e.g., metabolic syndrome, diabetes, cardiovascular disease).

As far as we are aware, the present systematic review and meta-analysis is the first to examine the prognostic value of fat and muscle mass in men with prostate cancer. In summary, increased levels of muscle mass and SAT and reduced VAT/SAT ratio rather than overall adiposity are important prognostic factors in men with prostate cancer, even when controlling for multiple confounding factors. Furthermore, we provide rationale for future prospective analyses investigating the impact of sarcopenia and changes in muscle mass during cancer treatment on prostate cancer outcomes, as well as the investigation of strategies such as exercise and nutritional interventions to improve survival in this population.

## Supplementary information

Supplementary material file
