# Supplementary material for: Associations of fat and muscle mass with overall survival in men with prostate cancer: a systematic review with meta-analysis
Source: Prostate Cancer Prostatic Dis. 2021 Aug 21;25(4):615–26. doi: 10.1038/s41391-021-00442-0 (PMC9705235; doi:10.1038/s41391-021-00442-0)
Supplement: Supplementary file 1 — Supplementary material file [file 41391_2021_442_MOESM1_ESM.docx]

**Supplementary Online Content**

Lopez et al., Associations of fat and muscle mass with overall survival in men with prostate cancer: A systematic review and meta-analysis

**eAppendix 1.** Search strategy.

**eAppendix 2.** Eligible references for full-text articles assessment.

**eTable 1.** Assessment of Study Quality using the Newcastle-Ottawa Quality Assessment Scale (NOS) for cohort studies.

**eFigure 1.** Contour-enhanced funnel plot for A) fat mass and B) muscle mass associations with overall survival in men with prostate cancer.

**Appendix 1.** Search strategy

*Search strategy for PubMed*

(Prostate Neoplasms [title/abstract] OR Neoplasms, Prostate [title/abstract] OR Neoplasm, Prostate [title/abstract] OR Prostate Neoplasm [title/abstract] OR Neoplasms, Prostatic [title/abstract] OR Neoplasm, Prostatic [title/abstract] OR Prostatic Neoplasm [title/abstract] OR Prostate Cancer [title/abstract] OR Cancer, Prostate [title/abstract] OR Cancers, Prostate [title/abstract] OR Prostate Cancers [title/abstract] OR Cancer of the Prostate [title/abstract] OR Prostatic Cancer [title/abstract] OR Cancer, Prostatic [title/abstract] OR Cancers, Prostatic [title/abstract] OR Prostatic Cancers [title/abstract] OR Cancer of Prostate [title/abstract]) AND (“body composition” [title/abstract] OR “body fat” [title/abstract] OR “fat mass” [title/abstract] OR “visceral adipose tissue” [title/abstract] OR “subcutaneous adipose tissue” [title/abstract] OR "muscle mass" [title/abstract] OR "lean mass" [title/abstract] OR “sarcopenia” [title/abstract] OR “skeletal muscle”[title/abstract]) AND (“mortality” [title/abstract] OR “survival” [title/abstract] OR “death” [title/abstract])

*Search strategy for CINAHL and Web of Science*

(prostate cancer or prostatic neoplasms or prostate carcinoma) AND (“body composition” OR “body fat” OR “fat mass” OR “visceral adipose tissue” OR “subcutaneous adipose tissue” OR "muscle mass" OR "lean mass" OR “sarcopenia” OR “skeletal muscle”) AND (mortality OR survival OR death)

*Search strategy for EMBASE*

(‘prostate cancer’) AND ('body composition' OR 'body fat' OR 'fat mass' OR 'intra-abdominal fat' OR 'subcutaneous fat' OR 'muscle mass' OR 'lean mass' OR sarcopenia OR ‘skeletal muscle’) AND (mortality OR survival OR death)

*Search strategy for Cochrane Library*

(“prostate cancer”) AND (“body composition” OR “body fat” OR “fat mass” OR “visceral adipose tissue” OR “subcutaneous adipose tissue” OR "muscle mass" OR "lean mass" OR “sarcopenia” OR “skeletal muscle”) AND (mortality OR survival OR death)

**eAppendix 2.** References from articles screened for eligibility in the full-text assessment.

1. McDonald AMS, Thomas A.Mayhew, David L.Cardan, Rex A.Bakder, Christopher B.Harris, David M.Fiveash, John B. (S010) Computed Tomography–Assessed Measures of Bone Mineral Density and Muscle Mass as Predictors of Survival in Men With Prostate Cancer. Oncology (08909091). 2016:5-.

2. McCall KCC, S. C.Huang, Y.Kohl, N. E.Tupper, T.Van den Abbeele, A. D.Zukotynski, K. A.Sweeney, C. J. [(18)F]-Fluorodeoxyglucose Positron Emission Tomography/Computed Tomography of LAPC4-CR Castration-Resistant Prostate Cancer Xenograft Model in Soft Tissue Compartments. Transl Oncol. 2015 Jun;8(3):147-53.

3. Mustian KMP, L.Darling, T. V.Palesh, O.Heckler, C. E.Morrow, G. R. A 4-week home-based aerobic and resistance exercise program during radiation therapy: a pilot randomized clinical trial. Journal of Supportive Oncology. 2009;7(5):158-67.

4. Ohwaki KE, F.Hattori, K. Abdominal obesity, hypertension, antihypertensive medication use and biochemical recurrence of prostate cancer after radical prostatectomy. European Journal of Cancer. 2015 Mar;51(5):604-9.

5. Barreto RK, Y.Matsumoto, T.Pin, F.Colston, K. C.Couch, K. E.O'Connell, T. M.Couch, M. E.Bonewald, L. F.Bonetto, A. ACVR2B/Fc counteracts chemotherapy-induced loss of muscle and bone mass. Scientific Reports. 2017 Oct;7.

6. Morris EVE, C. M. Adipokines, adiposity, and bone marrow adipocytes: Dangerous accomplices in multiple myeloma. Journal of Cellular Physiology. 2018 Dec;233(12):9159-66.

7. Gupta AP, A.Ayers, C.Beg, M. S.Lakoski, S. G.Vega, G. L.Grundy, S. M.Johnson, D. H.Neeland, I. J. An Analysis of Individual Body Fat Depots and Risk of Developing Cancer: Insights From the Dallas Heart Study. Mayo Clinic Proceedings. 2017 Apr;92(4):536-43.

8. Tsai HKDA, A. V.Sadetsky, N.Chen, M. H.Carroll, P. R. Androgen deprivation therapy for localized prostate cancer and the risk of cardiovascular mortality. Journal of the National Cancer Institute. 2007 Oct;99(20):1516-24.

9. Saigal CSG, J. L.Krupski, T. L.Hanley, J.Schonlau, M.Litwin, M. S.Urologic Dis Am, Project. Androgen deprivation therapy increases cardiovascular morbidity in men with prostate cancer. Cancer. 2007 Oct;110(7):1493-500.

10. Basaria S. Androgen deprivation therapy, insulin resistance, and cardiovascular mortality: an inconvenient truth. J Androl. 2008 Sep-Oct;29(5):534-9.

11. Fang LCM, G. S.Wallner, K. E. Androgen Deprivation Therapy: A Survival Benefit or Detriment in Men With High-Risk Prostate Cancer? Oncology-New York. 2010 Aug;24(9):790-7.

12. Mucksavage PM, C.Kutikov, A.Wein, A. J.Torigian, D.Malkowicz, S. B. Anthropometric differences in obese men with biochemical failure after radical retropubic prostatectomy. Urologic Oncology-Seminars and Original Investigations. 2012 Sep-Oct;30(5):590-5.

13. Farris MSC, K. S.Kopciuk, K. A.McGregor, S. E.Friedenreich, C. M. Anthropometric measurements and survival after a prostate cancer diagnosis. Br J Cancer. 2018 Feb 20;118(4):607-10.

14. Di Sebastiano KMM, M. A critical evaluation of body composition modalities used to assess adipose and skeletal muscle tissue in cancer. Appl Physiol Nutr Metab. 2012 Oct;37(5):811-21.

15. Kim HSM, D. M.Smith, M. R.Presti, J. C.Aronson, W. J.Terris, M. K.Kane, C. J.Amling, C. L.Freedland, S. J. A natural history of weight change in men with prostate cancer on androgen-deprivation therapy (ADT): results from the Shared Equal Access Regional Cancer Hospital (SEARCH) database. Bju International. 2011 Mar;107(6):924-8.

16. Wallstrom PB, A.Gullberg, B.Olsson, H.Wirfalt, E. A prospective Swedish study on body size, body composition, diabetes, and prostate cancer risk. British Journal of Cancer. 2009 May;100(11):1799-805.

17. O'Neill RFH, F.Murray, L. J.O'Sullivan, J. M.Cantwell, M. M. A randomised controlled trial to evaluate the efficacy of a 6-month dietary and physical activity intervention for patients receiving androgen deprivation therapy for prostate cancer. Journal of Cancer Survivorship. 2015 Sep;9(3):431-40.

18. Zakaria HM, Massie L, Basheer A, Elibe E, Boyce-Fappiano D, Shultz L, Lee I, Griffith B, Siddiqui F, Chang V. Application of Morphometrics as a Predictor for Survival in Patients with Prostate Cancer Metastasis to the Spine. World Neurosurg. 2018 Jun;114:e913-e919.

19. Stangl-Kremser J, Suarez-Ibarrola R, Andrea D, Korn SM, Pones M, Kramer G, Marhold M, Krainer M, Enikeev DV, Glybochko PV, Tamandl D, Shariat SF, Baltzer P. Assessment of body composition in the advanced stage of castration-resistant prostate cancer: special focus on sarcopenia. Prostate Cancer Prostatic Dis. 2020 Jun;23(2):309-315.

20. Miladinovic D, Cusick T, Mahon KL, Haynes AM, Cortie CH, Meyer BJ, Stricker PD, Wittert GA, Butler LM, Horvath LG, Hoy AJ. Assessment of Periprostatic and Subcutaneous Adipose Tissue Lipolysis and Adipocyte Size from Men with Localized Prostate Cancer. Cancers (Basel). 2020 May 28;12(6):1385. doi: 10.3390/cancers12061385. PMID: 32481537; PMCID: PMC7352157.

21. Williams GRC, YanjunKenzik, Kelly M.McDonald, AndrewShachar, Shlomit S.Klepin, Heidi D.Kritchevsky, StephenBhatia, Smita. Assessment of Sarcopenia Measures, Survival, and Disability in Older Adults Before and After Diagnosis With Cancer. JAMA Network Open. 2020;3(5):e204783-e.

22. Greenlee HU, J. M.LeBlanc, M.Ramsey, S.Hershman, D. L. Association between Body Mass Index and Cancer Survival in a Pooled Analysis of 22 Clinical Trials. Cancer Epidemiology Biomarkers & Prevention. 2017 Jan;26(1):21-9.

23. Lee SJK, N. C. Association Between Sarcopenia and Metabolic Syndrome in Cancer Survivors. Cancer Nursing. 2017 Nov-Dec;40(6):479-87.

24. Wu WL, X.Chaftari, P.Cruz Carreras, M. T.Gonzalez, C.Viets-Upchurch, J.Merriman, K.Tu, S. M.Dalal, S.Yeung, S. C. Association of body composition with outcome of docetaxel chemotherapy in metastatic prostate cancer: a retrospective review. PLoS One. 2015;10(3):e0122047.

25. Joung JYP, S. Association of body composition with survival and efficacy of first-line treatment in patients with castration-resistant prostate cancer. Journal of Clinical Oncology. 2020 Feb;38(6).

26. Karakiewicz PI. Association of Muscle Mass with Survival after Radical Prostatectomy in Patients with Prostate Cancer. Journal of Urology. 2019 Sep;202(3):531-.

27. Ghosh SH, D.Parma, D. L.Ramirez, A.Li, R. Association of obesity and circulating adipose stromal cells among breast cancer survivors. Molecular Biology Reports. 2014 May;41(5):2907-16.

28. Huelster HX, M.Hatcher, J.Avulova, S.Glaser, Z.Moses, K. Association of sarcopenia versus obesity with survival in metastatic or castrate-resistant prostate cancer. Journal of Urology. 2020 Apr;203:E562-E.

29. Cushen SJP, D. G.McDermot, R.O'Sullivan, K.MacEneaney, P.Daly, L.Ryan, A. M. Body composition as a predictor of chemotherapy toxicity in patients with metastatic prostate cancer treated with docetaxel. Proceedings of the Nutrition Society. 2015;74(OCE4).

30. Dickerman BAT, J. E.Valdimarsdottir, U. A.Giovannucci, E.Wilson, K. M.Aspelund, T.Tryggvadottir, L.Sigurdardottir, L. G.Harris, T. B.Launer, L. J.Gudnason, V.Markt, S. C.Mucci, L. A. Body fat distribution on computed tomography imaging and prostate cancer risk and mortality in the AGES-Reykjavik study. Cancer. 2019 Aug 15;125(16):2877-85.

31. Mallah KND, C. J.Rhee, A. C.Scardino, P. T.Kattan, M. W. Body mass index is weakly associated with, and not a helpful predictor of, disease progression in men with clinically localized prostate carcinoma treated with radical prostatectomy. Cancer. 2005 May;103(10):2030-4.

32. Poulsen MHF, M.Abrahamsen, B.Gerke, O.Walter, S.Lund, L. Bone health and body composition changes in men treated with androgen deprivation therapy for prostate cancer. Scandinavian Journal of Urology. 2017;51(220):26-7.

33. Thum TS, J. Breakthrough in cachexia treatment through a novel selective androgen receptor modulator?! Journal of Cachexia Sarcopenia and Muscle. 2011 Sep;2(3):121-3.

34. Flori NL, H.Amac, S.Clavie, B.Fallieres, A.Francioni, L.Vaille, A.Georges, G.Lacroix, C.Senesse, P. Cancer in the obese subject: Impact on survival and oncological management. Nutrition Clinique Et Metabolisme. 2017 Oct;31(4):303-8.

35. Azvolinsky A. Cancer Prognosis: Role of BMI and Fat Tissue. JNCI: Journal of the National Cancer Institute. 2014;106(6):dju177-dju.

36. van Kruijsdijk RCMvdG, Y.Peeters, P. H. M.Visseren, F. L. J.Second Manifestations, ARTerial Dis. Cancer Risk in Patients with Manifest Vascular Disease: Effects of Smoking, Obesity, and Metabolic Syndrome. Cancer Epidemiology Biomarkers & Prevention. 2013 Jul;22(7):1267-77.

37. Efstathiou JAB, K.Shipley, W. U.Hanks, G. E.Pilepich, M. V.Sandler, H. M.Smith, M. R. Cardiovascular Mortality After Androgen Deprivation Therapy for Locally Advanced Prostate Cancer: RTOG 85-31. Journal of Clinical Oncology. 2009 Jan;27(1):92-9.

37. Pascual EMS, M. M.Alba, A. B.Rico, E. B.Gomez, R. C.Tormo, F. B. Central Body Fat Mass Measured by Bioelectrical Impedanciometry But Not Body Mass Index Is a High-Grade Prostate Cancer Risk Factor. Urologia Internationalis. 2017;98(1):28-31.

38. Ziaran SG, F.Breza, J. Changes in body composition, development of metabolic syndrome (X) and pathological fractures in men on long-term androgen deprivation therapy. Urology. 2011;78(3):S148-S9.

39. Ziaran SG, F. M.Stefancik, J.Trebaticky, B.Breza, J. Changes in body composition, lipid profile, serum fibrinogen, serum fasting glucose and red blood cell count in men on long-term androgen deprivation therapy. European Urology, Supplements. 2010;9(6):631.

40. Ramalingam SS, D. J.Gupta, R.Healy, P.Wu, Y.George, D. J.Armstrong, A. J.Harrison, M. R. Changes in skeletal muscle cross sectional area (CSA) in patients with metastatic castration-resistant prostate cancer (mCRPC) treated with enzalutamide (ENZ). Journal of Clinical Oncology. 2016;34.

41. Il'yasova DC, L. H.Harris, T. B.Newman, A. B.Bauer, D. C.Satterfield, S.Kritchevsky, S. B. Circulating levels of inflammatory markers and cancer risk in the health aging and body composition cohort. Cancer Epidemiol Biomarkers Prev. 2005 Oct;14(10):2413-8.

42. Loppenberg BR, F.Brock, M.von Bodmann, C.Michels, C. J.Noldus, J.Palisaar, J. Clinical and histopathological parameters of prostate cancer. Influence of anthropometric indices. Urologe. 2015 Jan;54(1):22-7.

43. O'Donoghue NS, S.Aktas, A.Hullihen, B.Ayvaz, S.Estfan, B.Walsh, D. Clinical significance of weight changes at diagnosis in solid tumours. Supportive Care in Cancer. 2019 Jul;27(7):2725-33.

44. Ziaran SG, F. M.Sn, J. B. Complex Metabolic and Skeletal Changes in Men Taking Long-Term Androgen Deprivation Therapy. Clinical Genitourinary Cancer. 2013 Mar;11(1):33-8.

45. McDonald AMS, T. A.Mayhew, D. L.Cardan, R. A.Baker, C. B.Harris, D. M.Yang, E. S.Fiveash, J. B. CT Measures of Bone Mineral Density and Muscle Mass Can Be Used to Predict Noncancer Death in Men with Prostate Cancer. Radiology. 2017 Feb;282(2):475-83.

46. Sheikhbahaei SR, D. K.Rowe, S. P.Pienta, K. J. CT-based assessment of body composition following neoadjuvant chemohormonal therapy in patients with castration-naïve oligometastatic prostate cancer. Prostate. 2020 Dec 1.

47. Tewari RR, S.Natu, S. M.Dalela, D.Goel, A.Goel, M. M.Tandon, P. Diet, obesity, and prostate health: are we missing the link? J Androl. 2012 Sep-Oct;33(5):763-76.

48. Rogers JW, C.Gujral, D. Does body mass index or subcutaneous adipose tissue thickness affect interfraction prostate motion in patients receiving radical prostate radiotherapy? Journal of Radiotherapy in Practice. 2016 Dec;15(4):334-40.

49. Kiwata JLD, T. B.Schroeder, E. T.Dieli-Conwright, C. M. Effect of a supervised exercise intervention on sarcopenic obesity and metabolic syndrome in prostate cancer patients: a randomized pilot study. Cancer research. 2017;77(13).

50. Muñoz-Rodríguez JD, A.Rosado, M. A.Centeno, C.Parejo, V.Costa-Trachsel, I.Gallardo, E.Bonfill, T.García-Rojo, D.De Verdonces, L.Prats, J. Effect of muscle density in patients with metastatic prostate cancer administered androgen deprivation therapy. Endocrinol Diabetes Nutr. 2020 Aug 20.

51. Brondfield SCW, V. K.Koepfgen, K. M.Molina, A.Ryan, C. J.Small, E. J.Harzstark, A. L. Effects of 6 months of abiraterone acetate (AA) on muscle and adipose mass in men with metastatic castration-resistant prostate cancer (mCRPC). Journal of Clinical Oncology. 2012;30(15).

52. Rumble YT, M.Groome, A.Olden, S.Cradock, I.Chowdhury, S. Effects of a 12-week exercise programme on cardiovascular risk and body composition in men receiving androgen deprivation therapy for prostate cancer. Journal of Clinical Oncology. 2018;36(15).

53. Ileana EA, S.Albiges, L.Massard, C.Di Palma, M.Escudier, B. J.Fizazi, K.Loriot, Y. Effects of abiraterone acetate and enzalutamide on muscle and adipose mass in men with metastatic castration-resistant prostate cancer (mCRPC). Journal of Clinical Oncology. 2013;31(15).

54. Speed-Andrews AEC, K. S. Effects of exercise on quality of life and prognosis in cancer survivors. Current sports medicine reports. 2009;8(4):176‐81.

55. Nilsen TSR, T.Skovlund, E.Courneya, K. S.Langberg, C. W.Lilleby, W.Fosså, S. D.Thorsen, L. Effects of strength training on body composition, physical functioning, and quality of life in prostate cancer patients during androgen deprivation therapy. Acta Oncol. 2015 Nov;54(10):1805-13.

56. Xu WHQ, Y. Y.Wang, J.Wang, H. K.Wan, F. N.Zhao, J. Y.Zhang, H. L.Ye, D. W. Elevated CD36 expression correlates with increased visceral adipose tissue and predicts poor prognosis in ccRCC patients. Journal of Cancer. 2019;10(19):4522-31.

57. Galvao DC, P.Taaffe, D. R.Spry, N.Joseph, D.Chambers, S. K.Newton, R. U. Exercise and genitourinary cancer survivorship. Asia-Pacific Journal of Clinical Oncology. 2013;9:63.

58. Wall BAG, D. A.Fatehee, N.Taaffe, D. R.Spry, N.Joseph, D.Hebert, J. J.Newton, R. U. Exercise Improves VO_2max_ and Body Composition in Androgen Deprivation Therapy-treated Prostate Cancer Patients. Medicine and Science in Sports and Exercise. 2017 Aug;49(8):1503-10.

59. Metcalf MC, C.Hand, L.Stanley, A.Bechtel, M.Chalise, P.Isaacson, T.Sullivan, D. K.Klemp, J.Befort, C.Thrasher, J. B.Hamilton-Reeves, J. M. Feasibility of a weight management program tailored for overweight men with localized prostate cancer: A pilot study. Journal of Urology. 2017;197(4):e1242.

60. Demark-Wahnefried WN, J. W.Hunter, G. R.Rais-Bahrami, S.Desmond, R. A.Chacko, B.Morrow, C. D.Azrad, M.Fruge, A. D.Tsuruta, Y.et al.,. Feasibility outcomes of a presurgical randomized controlled trial exploring the impact of caloric restriction and increased physical activity versus a wait-list control on tumor characteristics and circulating biomarkers in men electing prostatectomy for prostate cancer. BMC cancer. 2016;16(1) (no pagination).

61. Uth JH, T.Schmidt, J. F.Christensen, J. F.Frandsen, C.Christensen, K. B.Helge, E. W.Brasso, K.Rorth, M.Midtgaard, J.Krustrup, P. Football training improves lean body mass in men with prostate cancer undergoing androgen deprivation therapy. Scandinavian Journal of Medicine & Science in Sports. 2014 Aug;24:105-12.

62. Antoun S, Bayar A, Ileana E, Laplanche A, Fizazi K, di Palma M, Escudier B, Albiges L, Massard C, Loriot Y. High subcutaneous adipose tissue predicts the prognosis in metastatic castration-resistant prostate cancer patients in post chemotherapy setting. Eur J Cancer. 2015 Nov;51(17):2570-7.

63. Versteeg KSB, SusanneBuffart, Laurien M.de van der Schueren, Marian A. E.Langius, Jacqueline A. E.Verheul, Henk M. W.Maier, Andrea B.Konings, Inge R. Higher Muscle Strength Is Associated with Prolonged Survival in Older Patients with Advanced Cancer. Oncologist. 2018;23(5):580-5.

64. Couderc ALM, X.Nouguerede, E.Rey, D.Schneider, S.Champsaur, P.Lechevallier, E.Lalys, L.Villani, P. Hosage: Sarcopenia in Older Patients Before and After Treatment with Androgen Deprivation Therapy and Radiotherapy for Prostate Cancer. Journal of Nutrition Health & Aging. 2020 Feb;24(2):205-9.

65. Delouya GT, D.Bhatnagar, S. R.Campeau, S.Saad, F.Taussky, D. Impact of adipose tissue on prostate cancer aggressiveness - analysis of a high-risk population. Horm Mol Biol Clin Investig. 2018 Nov 24;36(3).

66. Power DGC, S.McDermott, R.Lim, M. C. J.McEneaney, P.Daly, L.Griffin, B.Murphy, K. P.Ryan, A. M. Impact of body composition parameters on clinical outcomes in patients with metastatic castration-resistant prostate cancer treated with docetaxel. Journal of Clinical Oncology. 2015;33(15).

67. Kampman EV, A.van Duijnhoven, F. J.Winkels, R. M. Impact of Diet, Body Mass Index, and Physical Activity on Cancer Survival. Curr Nutr Rep. 2012;1(1):30-6.

68. Dorff TG, M.Quinn, D. I.Pinski, J.Schroeder, T.Groshen, S.Dieli-Conwright, C.Kiwata, J. Impact of resistance exercise on metabolic syndrome (MetS) parameters in men receiving androgen deprivation therapy (ADT) for prostate cancer. Annals of Oncology. 2017;28:v555.

69. Rosado MAM, J.Dominguez, A.García, D.Gallardo, E.Bonfill, T.Costa, I.Prera, A.Hannaoui, N.Gonzalez, J. L.Abad, C.Vicente, E.Centeno, C.Capdevila, M.De Verdonces, L.Parejo, V.Planelles, P.Ferran, A.Prats, J. Impact of sarcopenia on the global survival of patients with prostate cancer with metastatic debut treated with androgenic-deprivation therapy...European Society for Clinical Nutrition and Metabolism (ESPEN) 40th Congress, September 1-4, 2018, Madrid, Spain. Clinical Nutrition. 2018;37:S177-S.

70. Vora MA, J.Shanti, R. M.Veillon, D.Cotelingam, J.Coppola, D.Shackelford, R. E. Increased Nicotinamide Phosphoribosyltransferase in Rhabdomyosarcomas and Leiomyosarcomas Compared to Skeletal and Smooth Muscle Tissue. Anticancer Research. 2016 Feb;36(2):503-7.

71. Fischer SC, S.Green, A.McWilliam, A.Descamps, T.Oing, C.Gillessen, S. Influence of treatment with abiraterone and enzalutamide on development of sarcopenia in patients with metastatic castration resistant prostate cancer. European Urology Open Science. 2020;19:e879.

72. Guerrios-Rivera LH, L.Frank, J.De Hoedt, A.Beverly, D.Grant, D. J.Hoyo, C.Freedland, S. J. Is Body Mass Index the Best Adiposity Measure for Prostate Cancer Risk? Results From a Veterans Affairs Biopsy Cohort. Urology. 2017 Jul;105:129-35.

73. Tinson AC, A. S.Zajac, J.Grossmann, M. Lack of improvement in fat mass following cessation of androgen deprivation therapy; a 4 year case-control study. Clinical Endocrinology. 2017;86:14-5.

74. Thekkekara RJP, S.Yadav, U.Ahmed, A. T.Mullane, M. R.Batra, K. K.Lad, T. E.Kato, C.Psutka, S. P. Lean and fat-mass changes following upfront docetaxel compared to androgen deprivation monotherapy in metastatic castration-naïve prostate cancer. Journal of Clinical Oncology. 2018;36(15).

75. Chang SH, S. D.Contois, J. H.Strom, S. S.Yamamura, Y.Babaian, R. J.Troncoso, P.Scardino, P. S.Wheeler, T. M.Amos, C. I.Spitz, M. R. Leptin and prostate cancer. Prostate. 2001 Jan 1;46(1):62-7.

76. Gong YD, L. J.Liang, J. Link between obesity and cancer: role of triglyceride/free fatty acid cycling. European Review for Medical and Pharmacological Sciences. 2014 Oct;18(19):2808-20.

77. Braga-Basaria MM, D. C.Carducci, M. A.Dobs, A. S.Basaria, S. Lipoprotein profile in men with prostate cancer undergoing androgen deprivation therapy. International Journal of Impotence Research. 2006 Sep;18(5):494-8.

78. Yassin DJD, G.Hammerer, P. G.Yassin, A. A. Long-Term Testosterone Treatment in Elderly Men with Hypogonadism and Erectile Dysfunction Reduces Obesity Parameters and Improves Metabolic Syndrome and Health-Related Quality of Life. Journal of Sexual Medicine. 2014 Jun;11(6):1567-76.

79. Murphy RW, M.Perrine, M.Pawlowicz, M.Mourtzakis, M.Lieffers, J.Maneshgar, M.Bruera, E.Clandinin, M.Baracos, V.Mazurak, V. Loss of adipose tissue and plasma phospholipids: Relationship to survival in advanced cancer patients. Clinical Nutrition. 2010;29(4):482-7.

80. Koo KCY, Y. E.Rha, K. H.Chung, B. H.Yang, S. C.Hong, S. J. Low body mass index is associated with adverse oncological outcomes following radical prostatectomy in Korean prostate cancer patients. Int Urol Nephrol. 2014 Oct;46(10):1935-40.

81. Gocan AGB, D.Schindler, A. E.Rohr, U. D. Managing immunity in resistant cancer patients correlates to survival: results and discussion of a pilot study. Hormone Molecular Biology and Clinical Investigation. 2011 Nov;8(2):455-69.

82. Cocco PB, J. Mortality from cancer of the male reproductive tract and environmental exposure to the anti-androgen p,p'-dichlorodiphenyldichloroethylene in the United States. Oncology. 1998;55(4):334-9.

83. Lee JH, J. E.Jang, W. S.Ham, W. S.Rha, K. H.Choi, Y. D. Muscle characteristics obtained from computed tomography may be prognosticators in castration-resistant prostate cancer patients: A single-center analysis of 453 patients. Journal of Urology. 2020;203:e562.

84. Christensen JFJ, L. W.Andersen, J. L.Daugaard, G.Rorth, M.Hojman, P. Muscle dysfunction in cancer patients. Annals of Oncology. 2014 May;25(5):947-58.

85. Storer TWM, R.Travison, T. G. Muscle function, physical performance and body composition changes in men with prostate cancer undergoing androgen deprivation therapy. Asian J Androl. 2012 Mar;14(2):204-21.

86. Bylow KH, J.Mohile, S. G.Stadler, W. M.Sajid, S.Dale, W. Obese Frailty, Physical Performance Deficits, and Falls in Older Men with Biochemical Recurrence of Prostate Cancer on Androgen Deprivation Therapy: A Case-control Study. Urology. 2011 Apr;77(4):934-40.

87. Bilic I. Obesity and cancer. Periodicum Biologorum. 2014 Dec;116(4):355-9.

88. Baldi MM, L.Seardo, M. A.Calvetti, C.Maccario, M.Lanfranco, F. Obesity and prostate diseases: the endocrine link. Obesity and Metabolism-Milan. 2007 Sep;3(3):131-42.

89. Smith MR. Obesity and sex steroids during gonadotropin-releasing hormone agonist treatment for prostate cancer. Clin Cancer Res. 2007 Jan 1;13(1):241-5.

90. Nam GEK, Y. H.Hare, K.Jung, J. H.Park, Y. G.Lee, K. W.Rhee, E. J.Son, J. W.Lee, S. S.Kwon, H. K. S.Lee, W. Y.Yoo, S. J.Korean Soc Study, Obesity. Obesity Fact Sheet in Korea, 2018: Data Focusing on Waist Circumference and Obesity-Related Comorbidities. Journal of Obesity & Metabolic Syndrome. 2019 Dec;28(4):236-45.

91. Xu MC, Huelster HL, Hatcher JB, Avulova S, Stocks BT, Glaser ZA, Moses KA, Silver HJ. Obesity is Associated with Longer Survival Independent of Sarcopenia and Myosteatosis in Metastatic and/or Castrate-Resistant Prostate Cancer. J Urol. 2021 Mar;205(3):800-805.

92. Fowke JHM, S. S.Concepcion, R. S.Penson, D. F.Barocas, D. A. Obesity, body composition, and prostate cancer. BMC Cancer. 2012 Jan 18;12:23.

93. Smith MR. Osteoporosis and obesity in men receiving hormone therapy for prostate cancer. J Urol. 2004 Nov;172(5 Pt 2):S52-6; discussion S6-7.

94. Das R. Overview on obesity and cancer in diverse populations. Cancer Epidemiology Biomarkers and Prevention. 2015;24(10).

95. Hendry JP, A.Leung, H.Salji, M. Peri-prostatic fat volume predicts castration resistance in advanced prostate cancer. European Urology, Supplements. 2016;15(3):e846.

96. Huang HC, S.Li, W.Bai, P.Wu, X.Xing, J. Periprostatic Fat Thickness on MRI is an Independent Predictor of Time to Castration-resistant Prostate Cancer in Chinese Patients With Newly Diagnosed Prostate Cancer Treated With Androgen Deprivation Therapy. Clin Genitourin Cancer. 2019 Oct;17(5):e1036-e47.

97. Henning SMG, C.Gollapudi, K.Byrd, J. B.Liang, P.Li, Z.Grogan, T.Elashoff, D.Magyar, C. E.Said, J.Cohen, P.Aronson, W. J. Phase II prospective randomized trial of weight loss prior to radical prostatectomy. Prostate Cancer Prostatic Dis. 2018 Jun;21(2):212-20.

98. Crespo CJG-P, M. R.Smit, E.Lee, I. M.McGee, D.Muti, P.Figueroa Valle, N. R.Ramierez-Marrero, F. A.Freudenheim, J. L.Sorlie, P. Physical activity and prostate cancer mortality in Puerto Rican men. J Phys Act Health. 2008 Nov;5(6):918-29.

99. Morales-Rojas JSL, A.Pareja-Galeano, H. Physical exercise minimizes the toxic triad for cancer: physical inactivity, low fitness, and obesity. European Journal of Human Movement. 2016;36:1-35.

100. Wong JRG, Z.Merrick, S.Wilson, P.Uematsu, M.Woo, K.Cheng, C. W. Potential for higher treatment failure in obese patients: correlation of elevated body mass index and increased daily prostate deviations from the radiation beam isocenters in an analysis of 1,465 computed tomographic images. International Journal of Radiation Oncology Biology Physics. 2009 Sep;75(1):49-55.

101. McCarty MF. Potential utility of natural polyphenols for reversing fat-induced insulin resistance. Medical Hypotheses. 2005;64(3):628-35.

102. Sasaki T, Sugino Y, Kato M, Nishikawa K, Kanda H. Pre-treatment ratio of periprostatic to subcutaneous fat thickness on MRI is an independent survival predictor in hormone-naïve men with advanced prostate cancer. Int J Clin Oncol. 2020 Feb;25(2):370-376.

103. Morimoto MA, K.Hara, T.Yamaoka, M. Prevention of body weight loss and sarcopenia by a novel selective androgen receptor modulator in cancer cachexia models. Oncology Letters. 2017 Dec;14(6):8066-71.

104. Ikeda T, Ishihara H, Iizuka J, Hashimoto Y, Yoshida K, Kakuta Y, Takagi T, Okumi M, Ishida H, Kondo T, Tanabe K. Prognostic impact of sarcopenia in patients with metastatic hormone-sensitive prostate cancer. Jpn J Clin Oncol. 2020 Aug 4;50(8):933-939.

105. Bluethmann SMW, M.Wasserman, E.Chen, C.Zaorsky, N. G.Hohl, R. J.McDonald, A. C. Prostate cancer in Pennsylvania: The role of older age at diagnosis, aggressiveness, and environmental risk factors on treatment and mortality using data from the Pennsylvania Cancer Registry. Cancer Medicine. 2020 May;9(10):3623-33.

106. Møller HR, N.Van Hemelrijck, M.Larsen, S. B.Cuzick, J.Holmberg, L.Overvad, K.Tjønneland, A. Prostate cancer incidence, clinical stage and survival in relation to obesity: a prospective cohort study in Denmark. Int J Cancer. 2015 Apr 15;136(8):1940-7.

107. Sanft TL, L.Harrigan, M.Cartmel, B.Zhou, Y.Chagpar, A.Pusztai, L.Irwin, M. Randomized controlled trial of weight loss vs. usual care on telomere length in women with breast cancer: The lifestyle, exercise and nutrition (LEAN) study. Cancer Research. 2016;76(4).

108. Kashiwagi ES, M.Masaoka, H.Imada, K.Monji, K.Takeuchi, A.Inokuchi, J.Tatsugami, K.Eto, M. Relationship between body composition and hormone sensitivity for androgen deprivation therapy in patients with metastatic prostate cancer. Prostate Int. 2020 Mar;8(1):22-6.

109. Glass OKR, S.Harrison, M. R. Resistance Exercise Training in Patients With Genitourinary Cancers to Mitigate Treatment-Related Skeletal Muscle Loss. Clinical Advances in Hematology & Oncology. 2016 Jun;14(6):436-46.

110. Lønbro SO, K.Primdahl, H.Johansen, J.Overgaard, J. Resistance training and dietary supplements as intervention for regaining muscle mass following radiotherapy in head and neck cancer patients. Radiotherapy and Oncology. 2011;98:S42.

112. Mukherji DJP, C.Bianchini, D.Tunariu, N.Cassidy, A. M.Omlin, A. G.Sandhu, S. K.Attard, G.De Bono, J. S. Sarcopenia and altered body composition following abiraterone acetate (AA) and corticosteroid (C) treatment in men with castration-refractory prostate cancer (CRPC). Journal of Clinical Oncology. 2012;30(15).

113. Pezaro CM, D.Tunariu, N.Cassidy, A. M.Omlin, A.Bianchini, D.Seed, G.Reid, A. H. M.Olmos, D.de Bono, J. S.Attard, G. Sarcopenia and change in body composition following maximal androgen suppression with abiraterone in men with castration-resistant prostate cancer. British Journal of Cancer. 2013 Jul;109(2):325-31.

114. Khan MBC, J.Parshad, S.Emmenegger, U. Sarcopenia assessment in men with metastatic castration-resistant prostate cancer (mCRPC) undergoing radium 223 (Ra223) therapy. Annals of Oncology. 2020;31:S535.

115. Ohtaka A, Aoki H, Nagata M, Kanayama M, Shimizu F, Ide H, Tsujimura A, Horie S. Sarcopenia is a poor prognostic factor of castration-resistant prostate cancer treated with docetaxel therapy. Prostate Int. 2019 Mar;7(1):9-14.

116. Caram MVB, E. L.Englesbe, M. J.Terjimanian, M.Wang, S. C.Griggs, J. J.Couriel, D. Sarcopenia is associated with autologous transplant-related outcomes in patients with lymphoma. Leukemia & Lymphoma. 2015 Oct;56(10):2855-62.

117. Zakaria HML, J. T.Telemi, E.Chuang, M.Abouelleil, M.Wilkinson, B.Chandra, A.Boyce-Fappiano, D.Elibe, E.Schultz, L.Siddiqui, F.Griffith, B.Kalkanis, S. N.Lee, I. Y.Chang, V. Sarcopenia Predicts Overall Survival in Patients with Lung, Breast, Prostate, or Myeloma Spine Metastases Undergoing Stereotactic Body Radiation Therapy (SBRT), Independent of Histology. Neurosurgery. 2020 May;86(5):705-16.

118. Avulova S, Stocks BT, Glaser Z, Penson DF, Moses KA. Mp87-06 Sarcopenia Rather Than Obesity May Be Associated With Survival Among Patients With Metastatic Or Castrate Resistant Prostate Cancer. J Urology. 2018;199(4S):e1189-e.

119. Di Palma MP, F.Goldwasser, F.Toledano, A.Raynard, B. Sarcopeniais common and clinically underestimated in patient with advanced prostate cancer. Supportive Care in Cancer. 2018;26:S388.

120. Dalton JTT, R. P.Mohler, M. L.Steiner, M. S. Selective androgen receptor modulators for the prevention and treatment of muscle wasting associated with cancer. Current Opinion in Supportive and Palliative Care. 2013 Dec;7(4):345-51.

121. Hanson EDS, A. K.Sood, S.Ma, L.Francis, J. D.Goldberg, A. P.Hurley, B. F. Strength training induces muscle hypertrophy and functional gains in black prostate cancer patients despite androgen deprivation therapy. J Gerontol A Biol Sci Med Sci. 2013 Apr;68(4):490-8.

122. McDonald AMF, J. B.Kirkland, R. S.Cardan, R. A.Jacob, R.Kim, R. Y.Dobelbower, M. C.Yang, E. S. Subcutaneous adipose tissue characteristics and the risk of biochemical recurrence in men with high-risk prostate cancer. Urologic Oncology-Seminars and Original Investigations. 2017 Nov;35(11).

123. Lee JSL, H. S.Ha, J. S.Han, K. S.Rha, K. H.Hong, S. J.Chung, B. H.Koo, K. C. Subcutaneous Fat Distribution is a Prognostic Biomarker for Men with Castration Resistant Prostate Cancer. J Urol. 2018 Jul;200(1):114-20.

124. Mason RJ, Boorjian SA, Bhindi B, Rangel L, Frank I, Karnes RJ, Tollefson MK. The Association Between Sarcopenia and Oncologic Outcomes After Radical Prostatectomy. Clin Genitourin Cancer. 2018 Jun;16(3):e629-e636.

125. Gregg JRS, D. S.Childs, A.Moll, N.Ward, J. F.Kim, J.Daniel, C. R.Logothetis, C.Bathala, T.Davis, J. W. The Association of Peri-Prostatic Fat and Grade Group Progression in Men with Localized Prostate Cancer on Active Surveillance. The Journal of urology. 2020:101097JU0000000000001321.

126. Foulkes SJD, R. M.Fraser, S. F. The clinical importance of quantifying body fat distribution during androgen deprivation therapy for prostate cancer. Endocr Relat Cancer. 2017 Mar;24(3):R35-r48.

127. Buttigliero C, Vana F, Bertaglia V, Vignani F, Fiori C, Osella G, Porpiglia F, Tucci M, Scagliotti GV, Berruti A. The fat body mass increase after adjuvant androgen deprivation therapy is predictive of prostate cancer outcome. Endocrine. 2015 Sep;50(1):223-30.

128. Blauwhoff-Buskermolen SL, J. A. E.Becker, A.Verheul, H. M. W.de van der Schueren, M. A. E. The influence of different muscle mass measurements on the diagnosis of cancer cachexia. Journal of Cachexia Sarcopenia and Muscle. 2017 Aug;8(4):615-22.

129. Di Sebastiano KMM, M. The role of dietary fat throughout the prostate cancer trajectory. Nutrients. 2014 Dec 22;6(12):6095-109.

130. Teillac PB, A. V.Irani, J.Wirth, M. P.Zlotta, A. R. The role of luteinizing hormone-releasing hormone therapy in locally advanced prostate cancer and biochemical failure: considerations for optimal use. Clin Ther. 2005 Mar;27(3):273-85.

131. Saad FG, L. J. The Role of Testosterone in the Etiology and Treatment of Obesity, the Metabolic Syndrome, and Diabetes Mellitus Type 2. Journal of Obesity. 2011;2011.

132. Dalton JTB, K. G.Bohl, C. E.Hancock, M. L.Rodriguez, D.Dodson, S. T.Morton, R. A.Steiner, M. S. The selective androgen receptor modulator GTx-024 (enobosarm) improves lean body mass and physical function in healthy elderly men and postmenopausal women: results of a double-blind, placebo-controlled phase II trial. Journal of Cachexia Sarcopenia and Muscle. 2011 Sep;2(3):153-61.

133. Fowke JHM, S. S.Barocas, D. A. Total body composition and the association with high-grade prostate cancer. Cancer Research. 2011;71(8).

134. Lucas ARB, R. L.Fanning, J.Isom, S.Rejeski, W. J.Klepin, H. D.Kritchevsky, S. B. Trajectories in muscular strength and physical function among men with and without prostate cancer in the health aging and body composition study. Plos One. 2020 Feb;15(2).

135. Prado CMMA, S.Sawyer, M. B.Baracos, V. E. Two faces of drug therapy in cancer: drug-related lean tissue loss and its adverse consequences to survival and toxicity. Current Opinion in Clinical Nutrition and Metabolic Care. 2011 May;14(3):250-4.

136. Ohwaki KE, F.Hattori, K. Visceral adipose tissue measured by computed tomography and high-grade prostate cancer after radical prostatectomy. International Journal of Obesity. 2015 Nov;39(11):1659-61.

137. Pennington ZP, B.Ahmed, A. K.Goodwin, C. R.Verlaan, J. J.Sciubba, D. M. Visceral Fat Volume From Standard Preoperative CT is an Independent Predictor of Short-term Survival in Patients Undergoing Surgery for Metastatic Spine Disease. Clinical Spine Surgery. 2019 Jul;32(6):E303-E10.

138. Kim MSJ, J. Y. Visceral Obesity Assessment by MRI and Prostate Cancer Risk. Journal of Magnetics. 2019 Dec;24(4):698-703.

139. Ock CYO, D. Y.Lee, J.Kim, T. Y.Lee, K. H.Han, S. W.Im, S. A.Kim, T. Y.Bang, Y. J. Weight loss at the first month of palliative chemotherapy predicts survival outcomes in patients with advanced gastric cancer. Gastric Cancer. 2016 Apr;19(2):597-606.

140. Lee J, Heo JE, Jang WS, Ham WS, Rha KH, Choi YD. MP37-06-2003 Muscle Characteristics Obtained From Computed Tomography May Be Prognosticators In Castration-Resistant Prostate Cancer Patients: A Single-Center Analysis Of 453 Patients. Journal of Urology. 2020;203(Supplement 4):e562-e.

141. Conteduca V, Caffo O, Derosa L, Veccia A, Petracci E, Chiuri VE, Santoni M, Santini D, Fratino L, Maines F, Testoni S, De Giorgi U. Metabolic syndrome in castration-resistant prostate cancer patients treated with abiraterone. Prostate. 2015 Sep;75(12):1329-38.

**eTable 1.** Assessment of Study Quality using the Newcastle-Ottawa Quality Assessment Scale (NOS) for cohort studies.

|  | **SELECTION** | | | | **COMPARABILITY** | **OUTCOME** | | |  |
| --- | --- | --- | --- | --- | --- | --- | --- | --- | --- |
| **Study** | **Q1. Representativeness of the exposed cohort** | **Q2. Selection of the non-exposed cohort** | **Q3. Ascertainment of exposure** | **Q4. Demonstration that outcome of interest was not present at start of study** | **Q5a and b. Comparability of cohorts on the basis of the design or analysis** | **Q6. Assessment of outcome** | **Q7. Was follow-up long enough for outcomes to occur** | **Q8. Adequacy of follow up of cohorts** | **TOTAL STUDY SCORE**  **(Sum of black stars)** |
| Antoun et al., 2015 | ★ | ☆ | ☆ | ★ | ★★ | ★ | ★ | ★ | 7 |
| Buttigliero et al., 2015 | ★ | ☆ | ☆ | ★ | ☆☆ | ★ | ★ | ★ | 5 |
| Wu et al., 2015 | ★ | ☆ | ☆ | ★ | ★★ | ★ | ☆ | ☆ | 5 |
| Cushen et al., 2016 | ★ | ☆ | ☆ | ★ | ★★ | ★ | ★ | ★ | 7 |
| McDonald et al., 2016 | ★ | ★ | ★ | ★ | ☆★ | ★ | ★ | ★ | 8 |
| Mason et al., 2018 | ★ | ★ | ★ | ★ | ☆★ | ★ | ★ | ★ | 8 |
| Zakaria et al., 2018 | ★ | ☆ | ☆ | ★ | ☆★ | ★ | ☆ | ☆ | 4 |
| Ohtaka et al., 2019 | ★ | ★ | ★ | ★ | ☆★ | ★ | ★ | ★ | 8 |
| Pak et al., 2019 | ★ | ☆ | ☆ | ★ | ★★ | ★ | ★ | ★ | 7 |
| Di Bella et al, 2020 | ★ | ☆ | ☆ | ★ | ☆★ | ★ | ★ | ★ | 6 |
| Ikeda et al., 2020 | ★ | ★ | ★ | ★ | ★★ | ★ | ★ | ★ | 9 |
| Lee et al., 2020 | ★ | ☆ | ☆ | ★ | ★★ | ★ | ☆ | ☆ | 5 |
| Pak et al., 2020 | ★ | ☆ | ☆ | ★ | ★★ | ★ | ★ | ★ | 7 |
| Sasaki et al., 2020 | ★ | ☆ | ☆ | ★ | ☆★ | ★ | ★ | ★ | 6 |
| Stangl-Kremser et al., 2020 | ★ | ☆ | ☆ | ★ | ★★ | ★ | ★ | ★ | 7 |
| Xu et al., 2020 | ★ | ★ | ★ | ★ | ★★ | ★ | ★ | ★ | 9 |
| **TOTAL ITEM SCORE** | 16 | 5 | 5 | 16 | 9 and 14 | 16 | 13 | 13 |  |

**
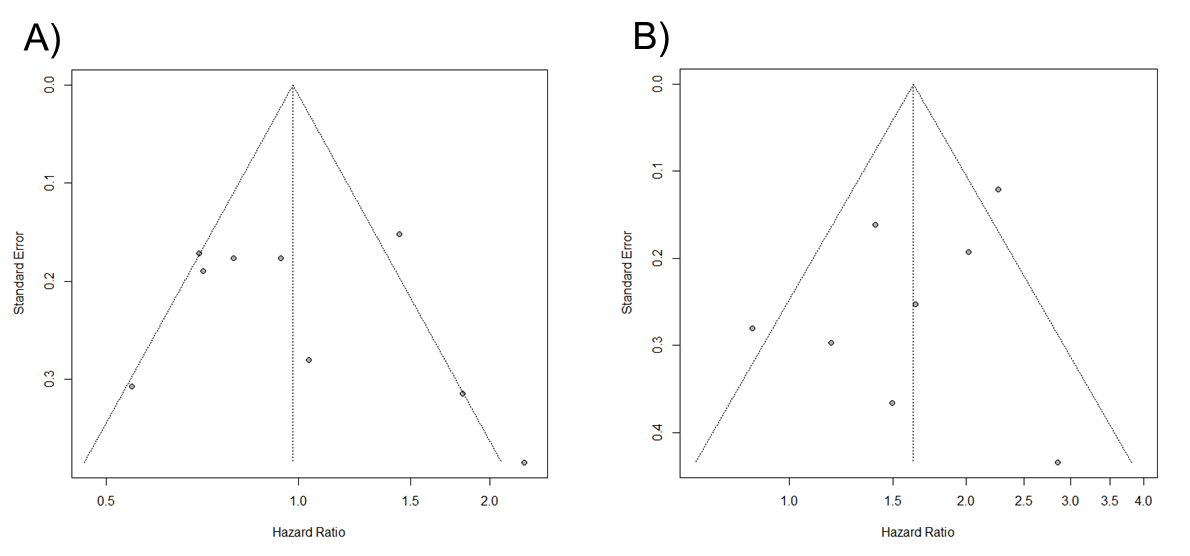
**

**eFigure 1.** Contour-enhanced funnel plot for A) fat mass and B) muscle mass associations with overall survival in men with prostate cancer.
